# Single-Cell Transcriptome Analysis of Chronic Antibody-Mediated Rejection After Renal Transplantation

**DOI:** 10.3389/fimmu.2021.767618

**Published:** 2022-01-17

**Authors:** Fanhua Kong, Shaojun Ye, Zibiao Zhong, Xin Zhou, Wei Zhou, Zhongzhong Liu, Jianan Lan, Yan Xiong, Qifa Ye

**Affiliations:** ^1^ Zhongnan Hospital of Wuhan University, Institute of Hepatobiliary Diseases of Wuhan University, Transplant Center of Wuhan University, Wuhan, China; ^2^ National Quality Control Center for Donated Organ Procurement, Hubei Key Laboratory of Medical Technology on Transplantation, Hubei Clinical Research Center for Natural Polymer Biological Liver, Hubei Engineering Center of Natural Polymer-Based Medical Materials, Wuhan, China; ^3^ The 3rd Xiangya Hospital of Central South University, Research Center of National Health Ministry on Transplantation Medicine Engineering and Technology, Changsha, China

**Keywords:** renal transplantation, chronic antibody-mediated rejection (cABMR), single-cell sequencing, end-stage renal disease, immunity, single-cell transcriptome

## Abstract

Renal transplantation is currently the most effective treatment for end-stage renal disease. However, chronic antibody-mediated rejection (cABMR) remains a serious obstacle for the long-term survival of patients with renal transplantation and a problem to be solved. At present, the role and mechanism underlying immune factors such as T- and B- cell subsets in cABMR after renal transplantation remain unclear. In this study, single-cell RNA sequencing (scRNA-seq) of peripheral blood monocytes (PBMCs) from cABMR and control subjects was performed to define the transcriptomic landscape at single-cell resolution. A comprehensive scRNA-seq analysis was performed. The results indicated that most cell types in the cABMR patients exhibited an intense interferon response and release of proinflammatory cytokines. In addition, we found that the expression of MT-ND6, CXCL8, NFKBIA, NFKBIZ, and other genes were up-regulated in T- and B-cells and these genes were associated with pro-inflammatory response and immune regulation. Western blot and qRT-PCR experiments also confirmed the up-regulated expression of these genes in cABMR. GO and KEGG enrichment analyses indicated that the overexpressed genes in T- and B-cells were mainly enriched in inflammatory pathways, including the TNF, IL-17, and Toll-like receptor signaling pathways. Additionally, MAPK and NF-κB signaling pathways were also involved in the occurrence and development of cABMR. This is consistent with the experimental results of Western blot. Trajectory analysis assembled the T-cell subsets into three differentiation paths with distinctive phenotypic and functional prog rams. CD8 effector T cells and γδ T cells showed three different differentiation trajectories, while CD8_MAI T cells and naive T cells primarily had two differentiation trajectories. Cell-cell interaction analysis revealed strong T/B cells and neutrophils activation in cABMR. Thus, the study offers new insight into pathogenesis and may have implications for the identification of novel therapeutic targets for cABMR.

## Introduction

End-stage renal disease (ESRD) is one of the major causes of concern for human health. At present, the most effective treatment is renal transplantation, however, rejection remains a major threat and is a primary independent risk factor affecting the long-term survival of the transplanted kidney (1, 2). In addition, cABMR is a serious threat to the long-term survival of the transplanted kidney which needs attention (3, 4). Although cABMR is the major cause of graft failure, its incidence, risk factors, and outcomes are largely unknown (5).

Currently, there are no effective drugs or preventive measures for cABMR treatment (6, 7). Some studies show that early intervention with ABMR can improve graft survival (8, 9). However, recent studies show that DSA-mediated advanced ABMR is highly resistant to a variety of therapeutic strategies (10, 11). Therefore, it is important to understand the host immune response during the disease to promote the design of the prognostic and early diagnostic markers, which can prevent the occurrence of cABMR in patients after kidney transplantation and to facilitate the design of appropriate appropriate therapeutic interventions to prevent graft loss.

The balance between the inflammatory and anti-inflammatory responses of the immune system determines the fate of the graft. The cytokine storm generated by the immune response after transplantation can lead to rejection and thus determines the survival of the graft. Therefore, the analysis of cytokine production may be important to elucidate the fate of the graft (12). The differentiation of B lymphocytes into plasma cells and the generation of donor-specific antibodies (DSA), which bind to human leukocyte antigen (HLA) or non-HLA molecules on endothelial cells causing endothelial cell damage, is the main mechanism of aABMR (13). Although B cells have considerable roles in the process of chronic rejection, new subsets of B cells have been identified in autoimmune diseases (14), cancers (15) and viral infections (16). They mediate immunomodulatory responses through various mechanisms broadly divided as interleukin-10-dependent- and interleukin-10-independent-pathways (12). Therefore, the effects and mechanisms of immune factors such as T- and B-cell subsets are not fully known in cABMR after renal transplantation. Information on the pathogenesis of T- and B-cell subsets in the early stage of cABMR are important for the early diagnosis of disease and development of new therapeutic targets for effective prolongation of graft survival time.

B lymphocytes differentiate into plasma cells and produce DSA, which bind to HLA or non-HLA molecules on endothelial cells and cause endothelial cell damage, which is the main mechanism of ABMR. Currently, DSA is a widely used biomarker for antibody mediated rejection (ABMR) (17). However, studies have shown that DSA can only correctly indicate 30-40% of ABMR patients, and some patients’ B lymphocytes are not activated to produce DSA (17, 18). Therefore, the effects and mechanisms caused by immune factors such as T and B cell subsets have not been fully revealed in cABMR after renal transplantation. Thus, it is of positive clinical value to explore the influence and mechanism of T and B cell subsets on the early onset of cABMR, to develop new therapeutic targets for the diagnosis and treatment of cABMR, and to effectively prolong the survival time of the graft.

In the present study, we performed scRNA-seq for an unbiased and comprehensive evaluation of the immunological responses in peripheral blood mononuclear cells (PBMCs) from patients with cABMR. Meanwhile, Western blot and qRT-PCR experiments were used to further confirm the results of single cell sequencing. Thus, such a high-resolution transcriptome map of immune cells during the occurrence and progression of cABMR after renal transplantation may provide implications for a better elucidation of the immunological panorama and pathogenic immune response of cABMR, and potential therapeutic strategies for clinical treatment of cABMR.

## Materials and Methods

### Sample Collection and Processing

Peripheral blood samples from 2 cABMR and 2 control patients after renal transplantation were collected from the Institute of Hepatobiliary Diseases of Wuhan University. The specimens were centrifuged at 400g for 5 min at 4°C, and the supernatant was discarded. Red blood cell lysate (SolarBio, R1010, China) was added and placed on ice for 15 min, followed by centrifugation at 400g at 4°C for 10min, to precipitate PBMCs. The supernatant was discarded and the PBMCs were resuspended by phosphate buffered saline to obtain a single-cell suspension.

All cABMR patients included in this project were diagnosed by the pathological section after renal transplantation, and they satisfied the Banff 2019 criteria (19). For chronic (active) ABMR the following 3 criteria for diagnosis were set:

1. Morphological evidence of chronic tissue injury, including 1 or more of the following: a. transplant glomerulopathy (cg>0) if there was no evidence of chronic TMA or chronic recurrent/*de novo* glomerulonephritis (included changes evident using electron microscopy (EM) alone) (cg1a). b. Severe peritubular capillary basement membrane multilayering (ptcml1; required EM). c. Arterial intimal fibrosis of new onset, excluding other causes; leukocytes within the sclerotic intima favor chronic ABMR if there was no prior history of TCMR, but are not required (19).
2. Evidence of current/recent antibody interaction with vascular endothelium, including 1 or more of the following: a. Linear C4d staining in peritubular capillaries or medullary vasa recta (C4d2 or C4d3 by IF using frozen sections, or C4d>0 by IHC in paraffin sections). b. At least moderate microvascular inflammation ([g+ptc] ≥2) in the absence of recurrent or *de novo* glomerulonephritis, although in the presence of acute TCMR, borderline infiltrate, or infection, ptc≥2 alone was not sufficient and g must be ≥1. c. Increased expression of gene transcripts/classifiers in the biopsy tissue is strongly associated with ABMR if thoroughly validated (19).
3. Identical to criterion 3 for active ABMR, including a strong recommendation for DSA testing whenever criteria 1 and 2 were met. Biopsies meeting criterion 1 but not criterion 2 with current or prior evidence of DSA (posttransplant) may be stated as ‘showing chronic ABMR’, however remote DSA was not considered for the diagnosis of chronic active or active ABMR (19).

### Microwell Single-Cell Sequencing

Single-cell suspensions with 1×10^5^ cells/mL in concentration in PBS (HyClone) were prepared. They were loaded onto microfluidic devices and scRNA-seq libraries were constructed according to the manufacturer’s (Singleron GEXSCOPE^®^) protocol using the GEXSCOPE^®^ Single-Cell RNA Library Kit (Singleron Biotechnologies) (20). Individual libraries were then diluted to 4nM and pooled for sequencing. These were sequenced on Illumina HiSeq X with 150 bp paired-end reads. The scRNA-Seq data are available at the Gene Expression Omnibus (https://www.ncbi.nlm.nih.gov/geo/. GEO accession number: GSE190329).

### Quality Control, Dimension-Reduction and Clustering

Raw reads were processed to generate gene expression profiles using a celescope1.3.0 pipeline. Briefly, after filtering read one without poly T tails, valid cell barcode and UMI was extracted. Adapters and poly A tails were trimmed (fastp V1) before aligning read two to GRCh38 with ensemble version 92 gene annotation (fastp 2.5.3a and featureCounts 1.6.2) (21). Reads with the same cell barcode, UMI and gene were grouped together to calculate the number of UMIs per gene per cell. The UMI count tables of each cellular barcode were used for further analysis. Cell type identification and clustering analysis using Seurat program (22). The Seurat program (http://satijalab.org/seurat/, R package,v.3.1.2) was applied for analysis of RNA-Sequencing data. UMI count tables were loaded into R using read table function. Mt-genes cutoff was set to 50. Any cells with UMI and genes number larger than 98% of the maximum UMI and genes number were not be used for further analysis. For each sample, we applied Harmony/v1.0 for batch correction and dimension reduction. Briefly, the dimensionality of the data was reduced by PCA (30 components, or 10 components for cell types with <5000 cells) first on the top 3000 (or 1000 for cell types with <5000 cells) most highly variable genes, followed by batch correction on sample ID. After dimension reduction by RunPCA, resolution 2 was used to do the clustering.

### Cell Type Annotation

**Figure 1:**
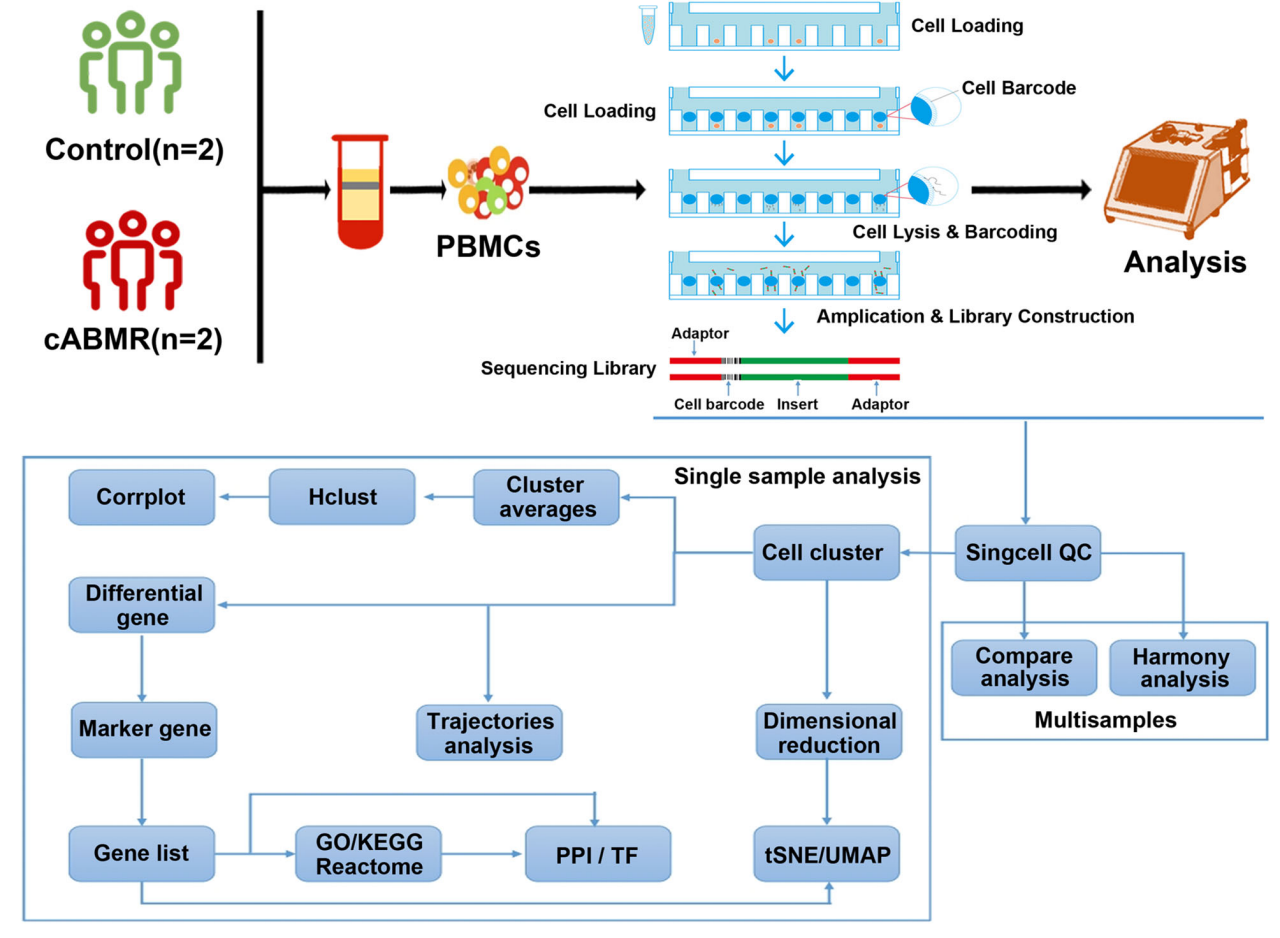
Schematic diagram of the overall design of the research on the acquisition of PBMCs and single-cell transcriptional profile analysis of normal patients and cABMR patients after renal transplantation. A total of 2 patients in the control group and 2 patients in cABMR were enrolled. After obtaining peripheral blood and extracting PBMCs, single cell suspension was obtained. To characterize the immunological features of patients with cABMR, we performed microwell-based scRNA-seq to evaluate the transcriptomic profiles of PBMCs。53,664,695 unique transcripts were obtained from 4 samples of PBMCs (a total of 39,285 cells; 23,633 (60%) from the control group and 15,652 (40%) from the cABMR group). We used the obtained sequencing data for quality control, dimension-reduction and clustering analysis, followed by cell type annotation, differential expression and GO/KEGG enrichment analysis, trajectory analysis and cell interaction analysis.

In order to determine cell types, we combined unsupervised clustering and differential expression to compare top differentially expressed genes with cell type specific expression known from literature. Through this approach, we confidently identified broad categories among all cells, and further delineated cellular subtypes by isolating subsets (through in silico “gating”) of broadly defined cell types and re-analyzing with the same approach. For broad cell type annotation shown in **Figure 1C**, low-resolution clustering was performed using the FindClusters function with resolution 2 with the first 20 PCs to generate clusters. Differential expression was performed using the FindAllMarkers function in Seurat with default parameters. To assign one of the 10 major cell types to each cluster, the marker genes we adopted were mainly from SynEcoSys single-cell database, which included more than 8800 marker genes (including 4541 human-derived marker genes, 4147 mouse-derived marker genes, and 388 rhesus monkey marker genes). 1369 cell types from 231 tissues/subtissues were involved in humans, 1079 cell types from 135 tissues/subtissues in mice, and 215 cell types from 19 tissues/subtissues in rhesus monkeys. At the same time, the data mainly comes from the latest single-celled sequencing and two big article databases [Cell Marker (http://biocc.hrbmu.edu.cn/CellMarker/index.jsp) and PanglaoDB (https://panglaodb.se/)]. Thus, we scored each cluster by the normalized expressions of the following canonical markers: T cells (CD3D, CD3E, TRBC1), NK cells (CD3D-, KLRD1, NKG7, KLRC1, FCGR3A), B cells (MS4A1, CD79A, CD79B), Neutrophils (LYZ, CSF3R, CXCR2, FCGR3B), Neutrophil progenitor cells(CAMP, LTF, LCN2, MPO, AZU1), Classical monocytes(LYZ, CD14, FCN1, VCAN, FCGR3A), Non-Classical monocytes(LYZ, FCN1, FCGR3A, CSF1R, CDKN1C), Dendritic cells(CD1C, CD1E, FCER1A, IL3RA, CLEC4C, LILRB4), Basophils(CLC, GATA2, CPA3, MS4A2), Patelets(PPBP, PF4, TUBB1). The final results were manually examined to ensure the correctness of the results and visualized by Uniform Manifold Approximation and Projection (UMAP). The 10 major cell types were chosen by initial exploratory inspection of the differentially expressed genes (DEGs) of each cluster combined with literature study. The DEGs were generated by Seurat FindMarkers function.

T cells and B cells subtype were determined using the ScaleData function in Seurat, and re-clustering at resolution (1.6) to obtain 4 and 2 clusters respectively. These clusters corresponded to the identities in **Figure 3B** and **Figure 5E**.

### Differential Expression and Enrichment Analysis

Differential gene expression (DEG) testing was performed using the FindMarkers function in Seurat with Wilcoxon test and p values were adjusted using Bonferroni correction. DEGs were filtered using a minimum log(fold change) of 0.25 and a maximum adjusted p value of 0.05 and were then ranked by average log(fold change) and FDR. Enrichment analysis for the functions of the DEGs was conducted using the clusterProfiler (v3.12.0) package. The gene sets were based on Gene Ontology terms and Kyoto Encyclopedia of Genes and Genomes (KEGG) pathways and analyzed by enrich KEGG and enrich GO. ggplot2 (Version:3.3.5) was used to draw the following result pictures (23).

### Trajectory Analysis

Monocle (Version 2.10.0) performed differential expression and time-series analysis for single-cell expression experiments. Size factor and dispersion were used to normalize data and select genes for clustering by estimateSizeFactors and estimateDispersions. Genes that weren’t highly expressed enough were not be used for clustering, since they would not give meaningful signal and would only add noise. Only genes likely to be informative of ordering of cells along the pseudotime trajectory would be selected (Highly Variable genes). Then, Monocle2 projected the data to 2 dimensions with DDRTree and did trajectory inference (orderCells) (24).

### Cell-Cell Interaction Score Calculation Using cellphoneDB

CellPhoneDB (Version 2.1.0) is a publicly available repository of curated receptors, ligands and their interactions and used to identify cellular communication across different celltypes. After extracted the counts matrix and cell annotation, “cellphonedb method statistical_analysis” this command finished the whole analysis. After that, “cellphonedb plot dot_plot”, “cellphonedb plot heatmap_plot” and several commands were used to calculate and draw the results. R packages: psych (Version 2.0.12), qgraph(Version 1.6.9), igraph (Version 1.2.6), purrr (Version 0.3.4) were used to draw count network (25).

### RNA Extraction and Quantitative Reverse Transcriptase-Polymerasechain Reaction

PMBC extracted from patients’ peripheral blood was treated with TRIzol reagent Invitrogen, America for 10 mins, centrifuged at 12,000 g at 4°C for 15 min. Then, the RNA to be suppressed was collected and mixed with isopropanol for isolation of the RNA. After RNA was obtained, RNA purity and concentration were analyzed using a Nanodrop 1000 spectrophotometer (Thermo Fisher, USA). CDNA was synthesized using a high capacity cDNA reverse transcription kit (Life Tec, America). The primers for genes were as follows:

MT-ND6:5′-ACGCCCATAATCATACAAAGCC-3′(Forward)
5′-TTGGTGCTGTGGGTGAAAGAGT-3′ (Reverse).
CXCL8: 5′- CTTGGCAGCCTTCCTGATTTC-3′(Forward)
5′- GGGGTGGAAAGGTTTGGAGTA-3′ (Reverse).
NFKBIA:5′- AAAGACGAGGAGTACGAGCAGAT-3′(Forward)
5′- CAGGTTGTTCTGGAAGTTGAGGA-3′ (Reverse).
ZFP36: 5′- CTCTGTCACAAGTTCTACCTCCAGG-3′(Forward)
5′- CCGGAGAAGCTGATGCTCTG-3′ (Reverse).
CXCR4:5′- GCCTTATCCTGCCTGGTATTGT-3′(Forward)
5′- AGGATGACTGTGGTCTTGAGGG-3′ (Reverse).
TNFAIP3: 5′- GCTGTTCAGCACGCTCAAGG-3′(Forward)
5′- TGGCCTTCTGAGGATGTTGC-3′ (Reverse).
S100A9:5′- GCTGGAACGCAACATAGAGACC-3′(Forward)
5′- CATTTGTGTCCAGGTCCTCCAT-3′ (Reverse).
NFKBIZ: 5′- GAGTCTGGTTGATACCATTAAGTGC-3′(Forward)
5′- CTGTTCGTTCTCCAAGTTCCG-3′ (Reverse).
DUSP1:5′- CTCAAAGGAGGATACGAAGCG-3′(Forward)
5′- GATGTCTGCCTTGTGGTTGTCC-3′ (Reverse).
JUN: 5′- CTGATAATCCAGTCCAGCAACG-3′(Forward)
5′- TTGAAGTTGCTGAGGTTTGCG-3′ (Reverse).
FOS:5′- GGGGCAAGGTGGAACAGTTA-3′(Forward)
5′- AGGTTGGCAATCTCGGTCTG-3′ (Reverse).
CCL4L2: 5′- TGAAGCTCTGCGTGACTGTCC-3′(Forward)
5′- GAGGCTGCTGGTCTCATAGTAATC-3′ (Reverse).

qRT-PCR was performed with 2X Universal SYBR Green Fast qPCR mix (Yeasen, China) on a LightCycler 96 system (Roche, America).

### Western Blot

Total protein was obtained from cells in radioimmunoprecipitation assay (RIPA) buffer (Beyotime, P0013B, Shanghai, China) containing phenylmethylsulfonyl fluoride (PMSF), aprotinin, and a phosphorylase inhibitor. The following primary antibodies were incubated for 12 h at 4°C: GAPDH (1:10000, Abclonal), MT-ND6 (1:1000, Abclonal), CXCL8 (1:1000, Proteintech), NFKBIZ (1:1000, Proteintech), JUN (1:1000, Proteintech), FOS (1:1000, Proteintech), NFKBIA (1:1000, Proteintech), NFκB (1:1000, Proteintech), DUSP1 (1:1000, Abclonal), The second antibody (1:5000, Abclonal) was incubated for 2 h at room temperature. Immunoreactive bands were developed using an Enhanced Chemiluminescence Detection Kit (Gen-view Scientific Inc., USA).

### Ethics Approval and Consent to Participate

This study was approved by the Medical Ethics Committee of Zhongnan Hospital of Wuhan University. All subjects provided informed consent and all experiments were conducted per the study protocol.

### Statistical Analysis

Statistical analyses included one-way ANOVA calculations and unpaired t test. SPSS24.0 software (SPSS, Inc., Chicago, IL, USA) was used for statistical analysis. P < 0.05 was considered statistically significant.

## Results

### Single-Cell Transcription for Characterization of Immunological Features in cABMR

To characterize the immunological features of patients with cABMR, we performed microwell-based scRNA-seq to evaluate the transcriptomic profiles of PBMCs from 2 cABMR patients and 2 control patients did not experience rejection after renal transplantation as controls. All patients in the control group were followed up without rejection after renal transplantation, and both patients in the cABMR group and the control group received the same anti-immune rejection drug (Mycophenolate mofetil, Tacrolimus (FK506) and Prednisone) strategy. The technical flow of the process is shown in **Figure 1**. Clinical characteristics of patients included in the study are detailed in **Supplementary Table 1** and **Supplementary Figure 1**. scRNA- sequencing was performed for each sample, and a unified single-cell analysis was performed as described in the previous section. 53,664,695 unique transcripts were obtained from 4 samples of PBMCs (a total of 39,285 cells; 23,633 (60%) from the control group and 15,652 (40%) from the cABMR group). Data preprocessing and quality control were performed to obtain transcriptome data (**Supplementary Figures 2A, B**).

### Differences in the Composition and Functions of Immune Cell Subsets in Patients With cABMR

**Figure 2:**
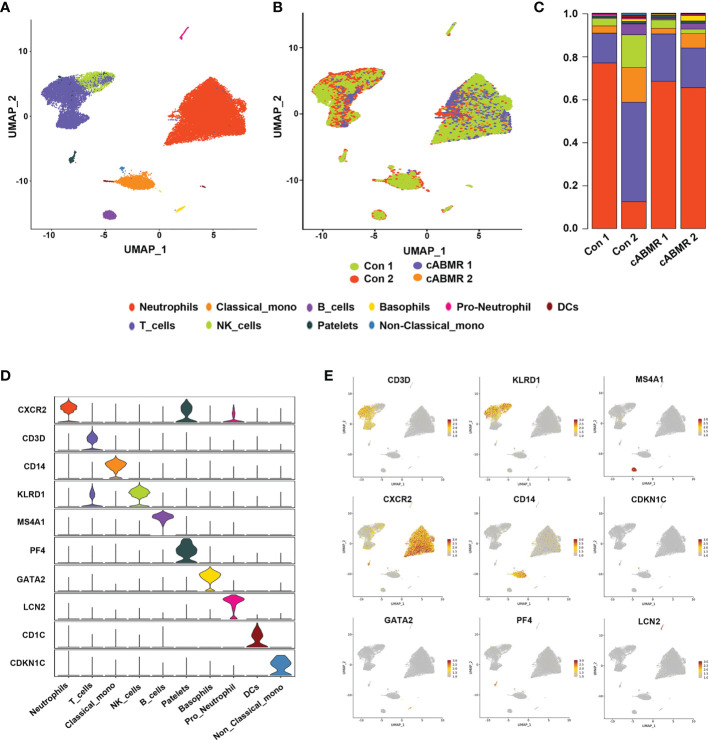
Differences in cell cluster composition between patients in Control group and cABMR group. **(A, B)** UMAP dimensional-reduction projection analysis of samples from the control group (n=2) and the cABMR group (n=2), **(A)** is stained by cell type source, and **(B)** is stained by group source, each dot corresponds to a single cell; **(C)** The mean proportion of each cell subsect from the Control group (n=2) and the cABMR group (n=2); **(D)** Violin plot of selected marker genes that identified the clusters generated by UMAP plotting. It was colored by different cell subtypes. **(E)** Canonical cell markers were used to label clusters by cell identity as represented in the UMAP plot. Data are colored according to expression levels and the legend is labeled in log scale.

To examine the differences in cell composition in the peripheral blood of cABMR patients and to compare them with the control group, we included 10 major cell types of PBMCs from each group based on scRNA-seq data. In cell projection clustering (UMAP) analysis, 10 major cell types and their transcriptome data were captured according to the markers of the typical genes of each cell cluster (**Figure 2A**), and the enrichment of different cell clusters in each sample (or group of samples) was calculated accordingly (**Figure 2B**). These cell clusters include T-cells, B-cells, NK cells, classical monocytes, non-classical monocytes, dendritic cells, basophils, platelets, neutrophil progenitors, and neutrophils. In **Figure 2C**, the bar graph depicts the percentage of cells aggregated in the peripheral blood of subjects in each patient. **Figures 2D, E** and **Supplementary Table 2** indicate the distribution of characteristic marker genes of these 10 cell clusters. Thus, we clearly defined the composition of peripheral blood cell clusters in patients after renal transplantation.

### 3 Immunological Features of T-Cell Subsets in Patients With cABMR

**Figure 3:**
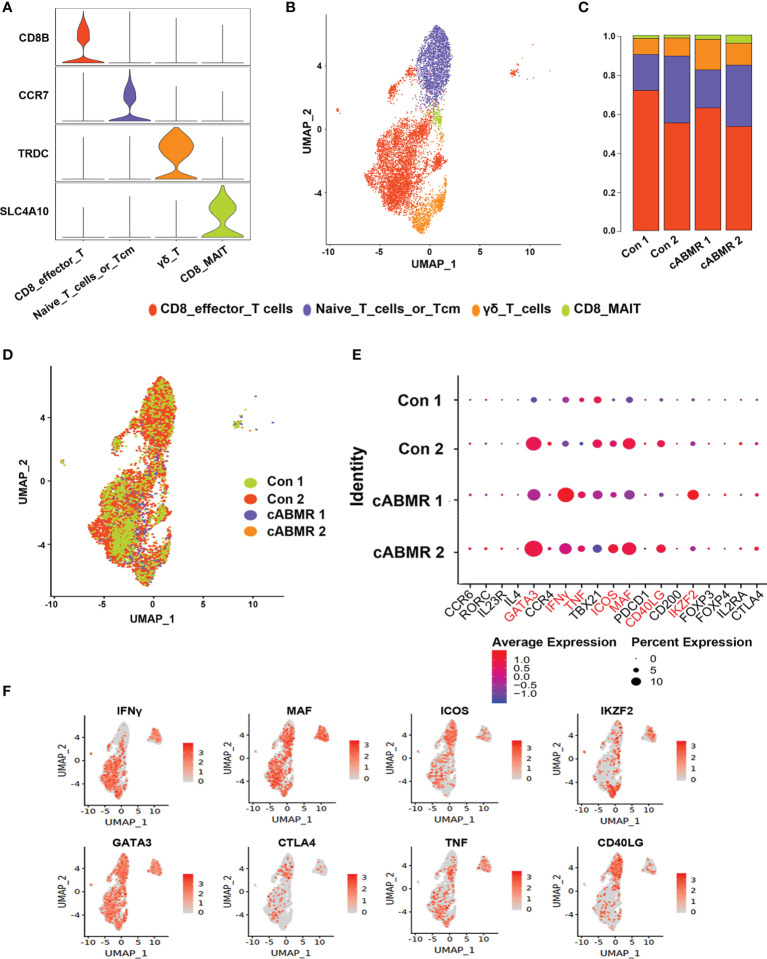
Immunological characteristics of T cell subsets. **(A)** The violin plot shows the distribution of selected marker genes in 4 subsets of T cells, with columns representing selected marker genes and rows representing subsets of the same color as in **(B)**; **(B)** UMAP dimensional-reduction projection analysis of samples from the control group and the cABMR group, which was stained by cell type source; **(C)** The proportion of T cell subsets from the Control (n=2) and cABMR (n=2) samples; **(D)** UMAP dimensional-reduction projection analysis of samples from the control group and the cABMR group, which was stained by sample source, each dot corresponds to a single cell; **(E)** DEGs node maps of T cells in the control and cABMR samples, the size of the dot indicates the proportion of expression, and the shade of color indicates the average expression amount; **(F)** Canonical cell markers were used to label clusters by cell identity as represented in the T cell UMAP plot. Data are colored according to expression levels and the legend is labeled in log scale.

To analyze the changes in T-cell subsets expressed in cABMR patients, four subtypes were obtained, including CD8 mucosa-associated invariant T (CD8_MAI T) cells, γδ T cells, CD8 effector T cells, and primary T cells/central memory T cells (**Figure 3B**), by labeling the genes typical of the T-cell subsets (**Figure 3A** and **Supplementary Table 3**). The relative proportions of the four T-cell subtypes in each patient was calculated (**Figure 3C**). Characteristics of T-cell subsets were analyzed, and the differences in the distribution of each subset between the control and the cABMR groups were assessed (**Figure 3D**). We also compared the representative DEGs in an undefined subpopulation of cells in T cells between the cABMR and the control groups. GATA3, IFNγ, TNF, ICOS, MAF, CD40LG, IKZF2, and CTLA4 were upregulated (**Figures 3E, F**).

**Figure 4:**
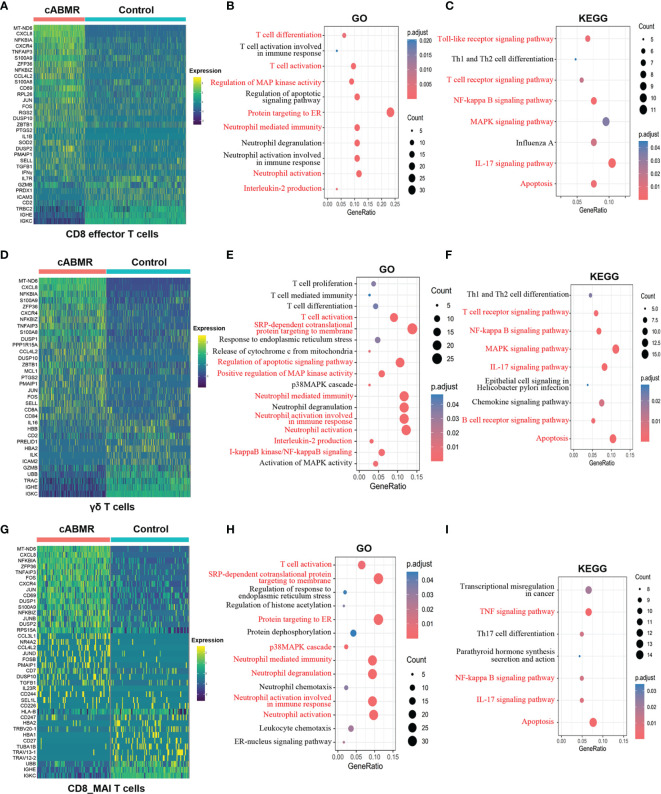
Differential genes expression, GO and KEGG enrichment analysis in T cell subsets. **(A)** Heat map of differential genes expression in CD8 effector T cells between control and cABMR group. Each column represents a group, and each row corresponds to a marker gene for each group; differential genes expression were filtered using a minimum log(fold change) of 0.25 and a maximum adjusted p value of 0.05 and were then ranked by average log(fold change) and FDR. **(B, C)** The GO **(B)** and KEGG **(C)** enrichment analysis of CD8 effector T cells between control and cABMR group; GO and KEGG terms are labeled with name and sorted by −log_10_ (P) value. A darker red indicates a smaller P value. The node size indicates the number of genes enriched. Interesting terms are labeled in red. **(D)** Heat map of differential genes expression in γδ T cells between control and cABMR group; **(E, F)** The GO **(E)** and KEGG **(F)** enrichment analysis of γδ T cells between control and cABMR group; **(G)** Heat map of differential genes expression in CD8_MAI T cells between control and cABMR group; **(H, I)** The GO **(H)** and KEGG **(I)** enrichment analysis of CD8_MAI T cells between control and cABMR group. GO and KEGG enrichment analysis revealed DEGs were mainly enriched in some biological processes and pathways in endothelial cells and glomerular endothelial cells respectively. Abbreviations were as follows: pct.exp, percentage of cells expressing gene; count, number of genes annotated to GO terms or KEGG pathway.

To further analyze the function of the T-cell subsets, we characterized DEGs in γδ T cells, CD8 effector T cells, and CD8_MAI T cells. Detailed information of DEGs per cell cluster of T-cells in cABMR and control groups is shown in **Supplementary Table 4**, and detailed information of GO and KEGG enrichment analyses is shown in **Supplementary Tables 5** **–** **7**. We found that DEGs in γδ T cells, CD8 effector T cells, and CD8_MAI T cells were up-regulated in the cABMR group which included MT-ND6, CXCL8, S100A9, NFKBIA, NFKBIZ, TNFAIP3, CXCR4, ZFP36, PMAIP1, DUSP10, FOS, JUN, and CCL4L2 (**Figure 4**). Most significant differences were obtained in the expression of MT-ND6, CXCL8, and NFKBIA. The role and mechanism of MT-ND6, a mitochondrial gene, in rejection has not yet been reported. The up-regulated expression of MT-ND6 may be related to mitochondrial oxidative stress. CXCL8 and S100A9 are involved in the activation of neutrophils. They are also involved in the activation of NF-κB signaling pathway including NFKBIA, TNFAIP3, and CCL4L2. NF-κB has been extensively studied in inflammation and tumor, but its role in antibody-mediated rejection is unknown. Interestingly, we also found that CXCR4, ZFP36, DUSP10, and FOS mediated the activation of MAPK signaling pathway. The MAPK signaling pathway regulates a variety of cellular functions, including proliferation, differentiation, apoptosis or survival, inflammation, and innate immunity (26). Thus, MAPK signaling pathway could be related to the occurrence of inflammation and immunity in cABMR.

Among tumor-related genes (CXCR4, FOS, DUSP10), FOS is a member of the FOS gene family and plays a regulatory role in cell proliferation, differentiation, and apoptosis (27). The mechanism of FOS in cABMR is less studied. CXCR4 leads to enhanced proliferation, migration, and invasion of tumor cells by binding to CXCL12 and activating various downstream signaling pathways (28). Some studies on the role of CXCR4 in immune rejection show that its antagonist can effectively reduce the intensity of rejection after transplantation (29, 30). These observations are consistent with our results. ZFP36, also known as adenosine triphosphate, is an RNA-binding protein (RBP) that is associated with cancer and has lower expression in some tumors (31). As a component of the MHC operon, ZFP36 is involved in the regulation of HLA-DQ gene processing and plays an important role in the regulation of immune response (32). GO and KEGG enrichment analyses (**Figure 4**) confirmed that both NF-κB and MAPK signaling pathways were involved in the activation of T-cells and antibody-mediated rejection; these may be related to the inflammatory response mediated by NF-κB and MAPK signaling pathways and their regulatory effects on immune cells need to be verified in future investigations. GO and KEGG enrichment analyses for downregulated genes are shown in **Supplementary Figure 3**.

GO enrichment analysis of γδ T cells and CD8 effector T cells (**Figures 4B, E**) showed that the biological process of IL-2 production and response to endoplasmic reticulum stress (ER stress). Secreted cytokines produced by IL-2-activated CD4+ and CD8+ T lymphocytes play an important role in the proliferation of T and B lymphocytes and the antigen-stimulated immune responses. KEGG enrichment analysis (**Figures 4C, F**) indicated the activation of apoptosis, IL-17 pathway, and Th1 and Th2 cell differentiation. IL-17 is a member of the IL-17 receptor family, consisting of five members (IL-17RA-E), which encodes for pro-inflammatory cytokines produced by activated T cells (33). This could explain our observations of a strong pro-inflammatory response in cABMR patients. Interestingly, we found the activation of the Toll-like receptor (TLR) signaling pathway and upregulated expression of related genes (CXCL8, NFKBIA, CCL4L2, JUN, FOS, IL1B, CCL4) in CD8 effector T cells. Activated TLR induces the expression of inflammatory cytokines and chemokines, and triggers cell-mediated immune responses. Therefore, the TLR-mediated signaling pathway is important in allograft rejection (34). In addition, DEGs in CD8_MAI T cells were also involved in gene enrichment of TNF and IL-17 signals and differentiation of Th17 cells (**Figures 4G–I**).

**Figure 5:**
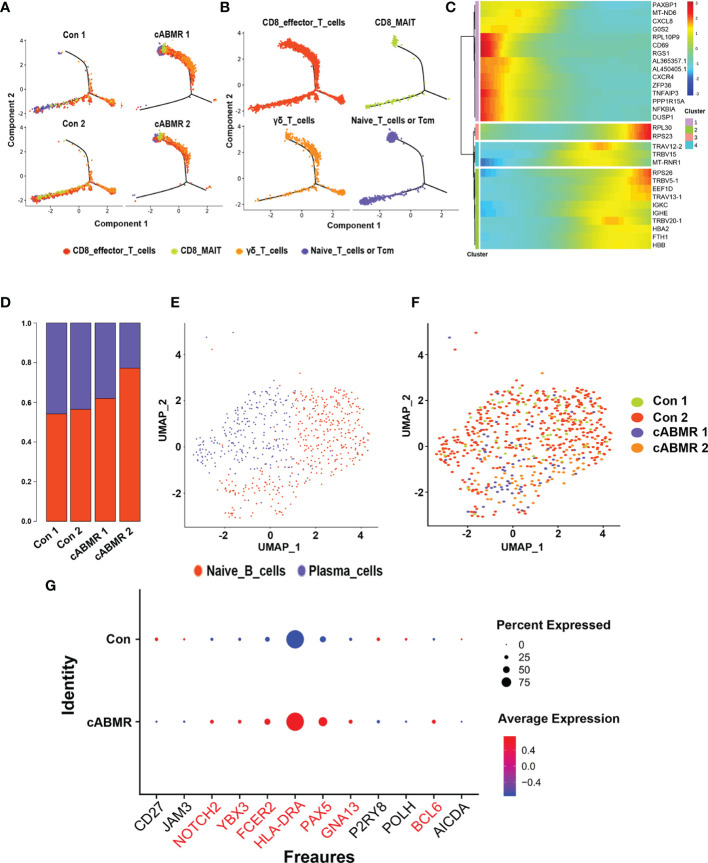
Trajectory analysis of T cell subsets and Immunological characteristics of B cell subsets. **(A, B)** T cell subsets (each subset after subdivision) pseudo time trajectory analysis; **(A)** is stained by group source, and **(B)** is stained by cell type source, each dot corresponds to a single cell; **(C)** Pseudotemporal gene-expression profiles of the DEGs in T cell subsets, excluding the ribosomal and mitochondrial genes; **(D)** The proportion of each subset of B cells in Control group (n=2) and cABMR group (n=2); **(E, F)** UMAP dimensional-reduction projection analysis of samples from the control group and the cABMR group, Among them, **(E)** is colored according to cell type, and **(F)** is colored according to sample source; **(G)** DEGs node maps of B cells in the control and cABMR samples.

To further examine the differentiation trajectories of T cell subsets, we performed monocle-based pseudotime analysis on our scRNA-seq data and created a developmental trajectory that traced the lineage specification of T cells as they matured. Three action trajectories were obtained in total. As shown in **Figure 5A**, the cABMR group showed a completely different trajectory of differentiation as compared to the control group. This indicated that the differentiation routes of T-cell subsets in cABMR were not consistent. To compare the differences in T-cell subsets, trajectory analysis of each subset was performed according to the four subsets defined previously (**Figure 5B**). CD8 effector T cells and γδ T cells showed three different differentiation trajectories, while CD8_MAI T cells and naive T cells primarily had two differentiation trajectories. In addition, we evaluated the variation in time-mimetic trajectories of differentially expressed genes. As shown in **Figure 5C**, we obtained a total of 4 differentially expressed gene clusters. In Cluster 1, a total of 15 DEGs (including MT-ND6, CXCL8, CD69, and PAXBP1) with the most significant differentially expressed genes were enriched. This was consistent with the results of GO and KEGG enrichment analysis.

### Immunological Features of B-Cell Subsets in Patients With cABMR

To evaluate the characteristics of B-cell subsets in cABMR, we labeled them according to the typical genes of B-cell subsets (**Supplementary Table 8**), and thus obtained two cell subtypes: the naive B cells and the plasma cells. **Figure 5D** shows the percentage of naive B-cells to plasma cells in 4 samples. To examine the characteristics of B-cell subsets, we assessed the distribution differences in each subset between the control group and the cABMR group (**Figures 5E, F**). By comparing the transcriptional profiles of cABMR and the control groups, we defined the representative marker genes of naive B cells and plasma cells. **Figure 5G** shows the overexpressed marker genes in B cells.

**Figure 6:**
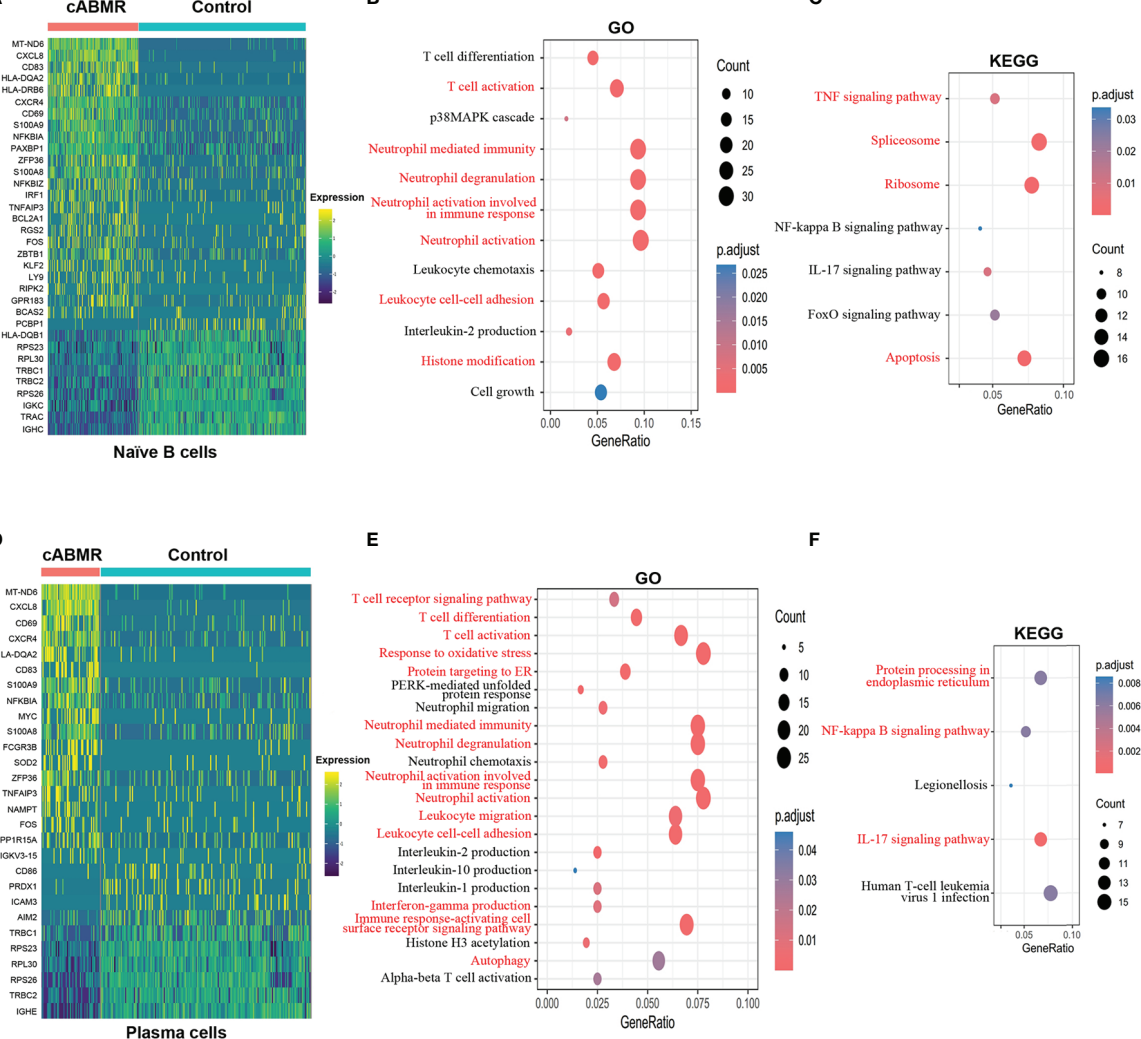
Differential genes expression, GO and KEGG enrichment analysis in B cell subsets. **(A)** Heat map of differential genes expression in Naïve B cells between control and cABMR group. Each column represents a group, and each row corresponds to a marker gene for each group. Differential genes expression differential genes expression were filtered using a minimum log(fold change) of 0.25 and a maximum adjusted p value of 0.05 and were then ranked by average log(fold change) and FDR. **(B, C)** The GO **(B)** and KEGG **(C)** enrichment analysis of Naïve B cells between control and cABMR group; GO and KEGG terms are labeled with name and sorted by −log_10_ (P) value. A darker red indicates a smaller P value. The node size indicates the number of genes enriched. Interesting terms are labeled in red. **(D)** Heat map of differential genes expression in Plasma cells between control and cABMR group; **(E, F)** The GO **(B)** and KEGG **(C)** enrichment analysis of plasma cells between control and cABMR group. Abbreviations were as follows: pct.exp, percentage of cells expressing gene; count, number of genes annotated to GO terms or KEGG pathway.

Next, we compared the changes in DEGs between naive B cells and plasma cells (**Figures 6A, D**). Details of DEGs in each cluster of B-cells in the cABMR and control groups are shown in **Supplementary Table 9**, and details of GO and KEGG enrichment analyses are shown in **Supplementary Tables 10**, **11**. DEGs analysis indicated that NFKBIA, CD69, CD83, TFAIP3, CXCR4, ZFP36, S100A8, S100A9, CXCL8, FOS, MT-ND6, and HLA-DQA2 in both naive B-cells and plasma cells were overexpressed in cABMR group (**Figure 6**). The expression differences of MT-ND6, CXCL8, and NFKBIA in all subsets of B cells were the most significant; exhibiting the same expression trend as that of T-cells. Compared to T-cells, CD69, CD83, and HLA-DQA2 were upregulated in B cells. CD69 is an early activation biomarker for T cell activation induction. Interestingly, CD69 also acts as a receptor for upregulated S100A8 and S100A9 in B-cells and forms S100A8/S100A9-CD69 complex through glycation, which is involved in regulating regulatory T-cells (Treg) differentiation (35). However, the role of CD69 in B cells remains largely unknown. In addition, there are limited studies on the role of CD83 in antibody-mediated rejection. CD83 may be involved in antibody-mediated rejection and could regulate adaptive immune response and activate T- and B-lymphocytes. Both HLA-DQA2 and HLA-DRB6 belong to a subclass of MHC (36) and play an important role in the peptide loading of MHC class II molecules. These are expressed in B lymphocytes, dendritic cells, and macrophages and are used to present antigenic peptides on the cell surface for recognition by the CD4 T cells.

Furthermore, we analyzed the functional characteristics of B-cell subsets. In both GO (**Figure 6B** and **Supplementary Figure 4A**) and KEGG enrichment analyses of naive B-cells (**Figure 6C** and **Supplementary Figure 4B**), we found that DEGs are enriched during the biological processes of T-cell activation and neutrophils activation. Additionally, DEGs were also enriched in IL-2 production and p38MAPK cascade. In cABMR, naive B-cells are involved in the activation of TNF, IL-17, NF-κB, and FoxO signaling pathways. In TNF signaling, NFKBIA, TNFAIP3, IRF1, FOS, PTGS2, MAP3K8, AKT2, TNFRSF1B, CFLAR, ATF4, etc. were upregulated. Genes involved in the regulation of IL-17 signaling, including CXCL8, S100A9, NFKBIA, S100A8, TNFAIP3, FOS, JUND, PTGS2, and FOSB, were also up-regulated in cABMR; Interestingly, we found activation of FoxO signaling in naive B-cells and its involvement in cABMR. FOXOs are widely involved in regulating various cell functions, including cell differentiation, apoptosis, cell proliferation, DNA damage and repair, and act as mediators of oxidative stress (37). However, the role and mechanism of FoxO signaling in cABMR remains unknown.

In plasma cells, GO (**Figure 6E** and **Supplementary Figure 4C**) and KEGG enrichment analyses (**Figure 6F** and **Supplementary Figure 4D**) showed that DEGs not only mediated T-cell activation and neutrophil activation but also were enriched in the biological processes of ER stress, which suggested that ER stress was involved in cABMR after renal transplantation. Although the mechanism of ER stress in solid organ transplantation is largely unclear, there is increasing evidence that ER stress plays an important role in mediating allograft injury (38). Interestingly, we also found an enrichment of DEGs related to oxidative stress in plasma cells in the GO enrichment analysis (**Figure 6E**). Moreover, ER-related differential genes were upregulated in ABMR. These interfere with the degradation of secreted proteins and transmembrane proteins in the process of ischemia-reperfusion injury or rejection of transplanted organs (39), which could explain our findings.

### qRT-PCR and Western Blot Confirmed the Activation of MAPK and NFκB Signaling Pathways

**Figure 7:**
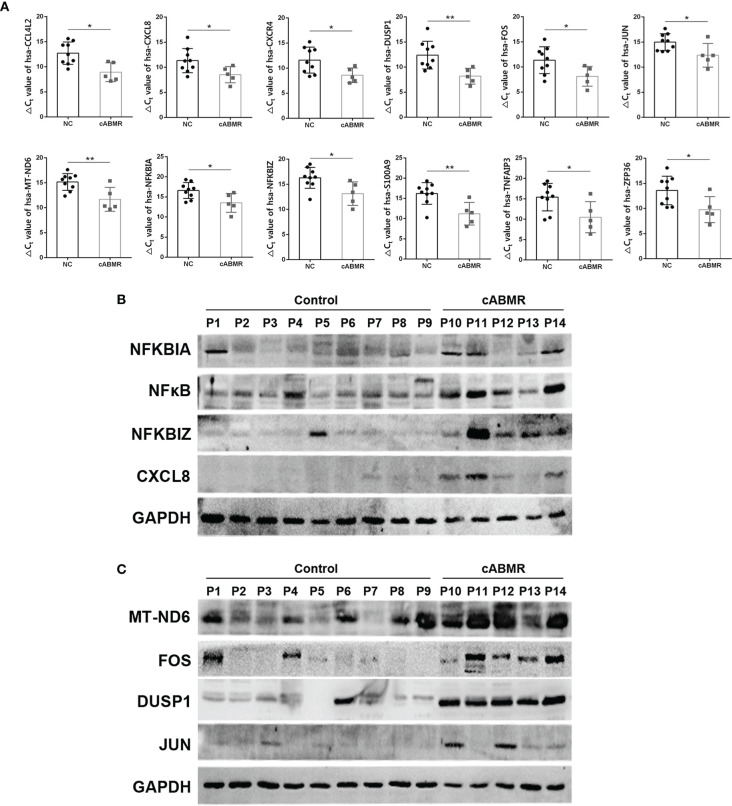
qRT-PCR and Western blot Experiment were performed to verify the changes of differentially expressed genes in cABMR patients. **(A)** qRT-PCR verified that MT-ND6, CXCL8, NFKBIA, ZFP36, CXCR4, TNFAIP3, S100A9, NFKBIZ, DUSP1, JUN, FOS, CCL4L2 were up-regulated in peripheral blood of cABMR patients. **(B)** Western blot verified that NFκB signaling pathway related protein expression was up-regulated. **(C)** Western blot verified that MAPK signaling pathway related protein expression was up-regulated. Data are shown as the mean ± SD (*P < 0.05, **P < 0.01 vs. control).

To further verify the results of the above differentially expressed genes, GO and KEGG enrichment analysis, a total of 9 control patients(P1-P9) and 5 cABMR patients(P10-P14) were enrolled and their peripheral blood was collecteds. The patient demographics of most relevant information for renal transplant patients as show in **Supplementary Table 1**. After PBMC separation, protein and total RNA were extracted. qRT-PCR showed that MAPK and NFκB signaling pathways were activated in cABMR patients. As shown in **Figure 7A**, IL-17 signaling Pathway related genes (CXCL8/NFKBIA/TNFAIP3/S100A9/JUN/FOS) were up-regulated in peripheral blood of patients with cABMR. In addition, we confirmed that MT-ND6, CCL4L2, CXCR4, NFKBIZ, DUSP1 and ZFP36 were up-regulated by qRT-PCR (**Figure 7A**). △Ct values corresponding to all genes are shown in **Supplementary Table 12**. Therefore, qRT-PCR results showed that screening potential molecular markers from peripheral blood of cABMR patients by single-cell sequencing is feasible and effective. Meanwhile, activation of MAPK and NFκB signaling pathways was found in T and B cell subpopulations, which was confirmed by Western blot assay. As shown in **Figure 7B**, NFκB signaling pathway related proteins (NFKBIA/NFκB/NFKBIZ/CXCL8) were up-regulated in cABMR patients. In addition, MAPK signaling pathway related proteins (FOS/DUSP1/JUN) were also up-regulated in cABMR patients (**Figure 7C**). This is consistent with our single cell sequencing findings above.

### Comparative Analysis to Identify Cell-Cell Crosstalk in cABMR Through Ligand-Receptor Interactions

**Figure 8:**
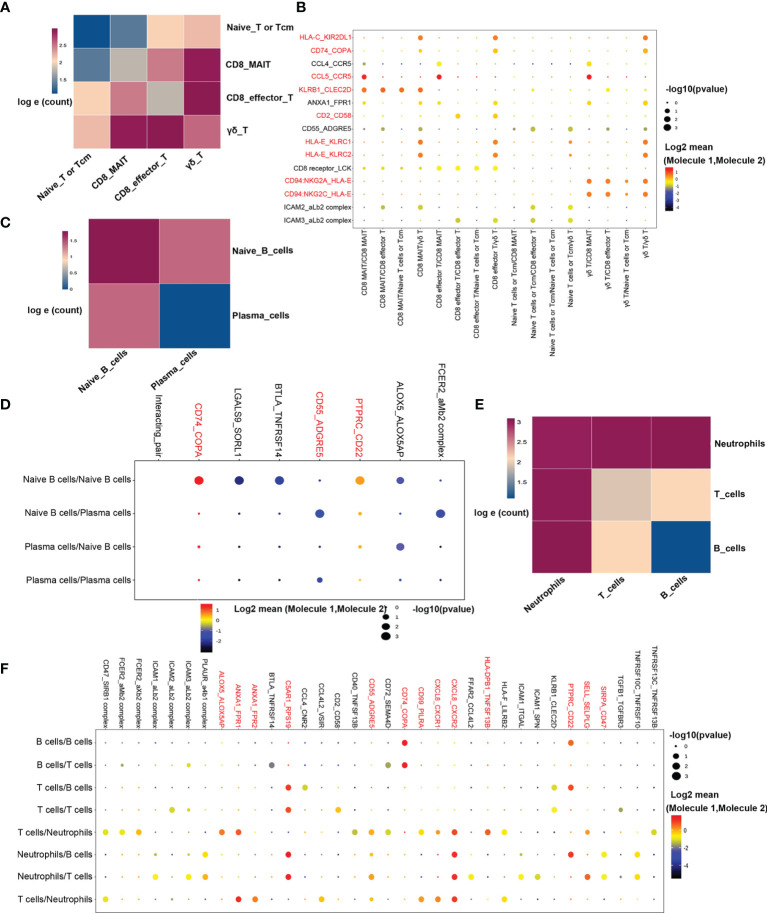
Possible ligand-receptor interactions between different cell types in PBMCs in patients with cABMR. **(A)** Schematic diagram of the strength of ligand-receptor interactions between T cell subsets. The numbers in the legend represent the log e conversion of the logarithm of the ligand between the two cell types. Darker colors indicate more logarithms of the interaction. **(B)** Representative ligand-receptor signaling axes interactions between T cell subsets. The size of the dot represents the expression proportion, and the color depth represents the average expression amount; **(C)** Schematic diagram of the ligand-receptor interaction intensity between naïve B cells and plasma cells; **(D)** Representative ligand-receptor signaling axes interactions between Naïve B cells and Plasma cells; **(E)** Schematic diagram of ligand-receptor interaction strength between T cells, B cells, and neutrophils; **(F)** Representative ligand-receptor signaling axes interactions between T cells, B cells, and neutrophils.

In transcriptome analysis of T-cells and B-cells, we found consistent activation of T-cells, B-cells, and neutrophils using GO and KEGG enrichment analyses. These findings indicated that the interaction between cells could be involved in the occurrence and development of cABMR. Thus, examining the interplay of cell-cell communication and signaling networks may provide novel insights into the discovery of new therapeutic targets for cABMR. **Figure 8** illustrates the potential interactions between receptors and ligands in different types of immune cells. First, we evaluated the interactions among T-cell subsets. **Figure 8A** shows the strength of interactions among T-cell subsets. Among them, the interactions among CD8_MAI T cells, CD8 effector T cells, and γδ T cells were the strongest. Analysis of the intercellular interactions showed that the CCL5-CCR5 signaling axis of the chemokine response pathway was the strongest in γδ T cells-CD8_MAI T cells, CD8 effector T cells-CD8_MAI T cells, and within CD8_MAI T cells (**Figure 8B**). γδ T cells activate other subsets *via* the CD94:NKG2A/C-HLA-E signaling axis. Thus, γδ T cells may participate in the pathogenesis of cABMR and induce damage to other cells by activating CD8+ T cells. The CD2-CD58 signaling axis also has an effect on CD8 effector T cells and γδ T cells. Other signaling axes, such as CD74-COPA, KLRB1-ClEC2D, and HLAE-KLRC1/2, also participated in the activation of T-cell subsets and could promote the occurrence and development of cABMR.

The strength of interaction between subsets of B-cells is shown in **Figure 8C**. **Figure 8D** shows the information of interaction molecules between each subpopulation of B-cells. In naive B-cells, we observed activation of the CD74-COPA and the PTPRC-CD22 signaling axes. The PTPRC-CD22 signal axis is an important regulator of T-cell and B-cell antigen receptor signal transduction. It acts through direct interaction with the antigen receptor complex or through activation of various Src- family kinases for antigen receptor signaling. CD55-ADGRE5 signaling axis is also involved in signal transduction between naive B cells and plasma cells and plays a role in cell adhesion as well as leukocyte recruitment, activation, and migration.

In the GO and KEGG enrichment analyses, biological processes activated by neutrophils played an important role in the occurrence of cABMR. Therefore, the underlying mechanism of the interaction between T/B cells and neutrophils was further analyzed (**Figure 8E**). Notably, the CD74-COPA and the PTPRC-CD22 signaling axes are not only activated in B-cells but also play an important role in signal transduction between T/B cells and neutrophils (**Figure 8F**). In addition, CXCL8, the most enriched gene, was involved in neutrophils activation through the CXCL8-CXCR1/2 signaling axis (**Figure 8F**). In addition, the ANXA1-FPR1/2 signaling axis is also involved in the activation of neutrophils, and we report for the first time, the involvement of ANXA1-FPR1/2H and C5AR1-RPS19 signaling axes in cABMR (**Figure 8F**). Mechanisms underlying the ANXA1-FPR1/2 and C5AR1-RPS19 signal axes are unclear. Most of the mechanistic studies focus on their influence on the platelet function (such as platelet adhesion, activation, aggregation, and thrombosis) (40). Thus, studies investigating the mechanisms of cABMR are important.

## Discussion

In this study, we comprehensively generated the profiles of distinct cell types and gene expression in peripheral blood specimens using scRNA-seq in cABMR patients. We also verified that scRNA-seq analysis of PBMCs in cABMR patients was a feasible and effective technique. The best treatment for cABMR is its prevention (6). The knowledge of roles and mechanisms of T- and B-cell subsets in the early onset of cABMR are of great clinical value for the diagnosis, potential new therapeutic targets, and effective prolongation of graft survival time.

We reported the upregulation of several genes (such as NFKBIA, NFKBIZ, TNFAIP3, CXCR4, ZFP36, PMAIP1, DUSP10, and S100A9) in T-cells for the first time. DEGs are involved in IFN response and pro-inflammatory cytokine production in cABMR patients. IFNγ is a cytokine that plays an important role in tissue homeostasis, immune and inflammatory responses, and tumor immune monitoring. IFNγ is produced mainly by immune cells, including innate-like lymphocyte populations, such as natural killer (NK) cells and innate lymphoid cells (ILCs), and adaptive immune cells, such as T helper 1 (TH1) cells and CD8+ cytotoxic T lymphocytes (CTLs) (41). TNF is one of the pro-inflammatory cytokines and a member of the tumor necrosis factor superfamily (42). TNF can bind and act through its receptors, TNFRSF1A/TNFR1 and TNFRSF1b/TNFBR (43). This cytokine is involved in the regulation of a variety of biological processes, including cell proliferation, differentiation, apoptosis, lipid metabolism, and coagulation. In addition, the up-regulation of GATA3, ICOS, IKZF2, and CTLA4 promotes the development and differentiation of T cells. Interestingly, the up-regulation of CD40LG indicates that T-cells regulate the function of B cells by the adsorption of CD40 on the surface of B-cells by CD40LG and activate B-cell-mediated rejection. Therefore, relatively strong interferon and overall proinflammatory responses were observed in cABMR. In addition to the up-regulation of NFKBIA, TNFAIP3, CXCR4, ZFP36, S100A8, S100A9, CXCL8, FOS, and MT-ND6, the expression of CD69, CD83, and HLA-DQA2 was also found in B- cells. CD69 regulates the adaptive immune response through a variety of mechanisms, including enhancing Treg inhibitory activity, maintaining the balance between Th17 and Treg by preventing Th17 differentiation, and controlling T-cell development, maturation, and departure (44). CD83 expression has been identified in several activated immune cells, including B and T lymphocytes, monocytes, dendritic cells, microglia, and neutrophils. In addition, CD83 is an essential factor in T- and B- lymphocyte differentiation, their formation, and maintenance of tolerance (45). The DEGs in this study were mainly enriched in neutrophils activation, interferon response, overall proinflammatory response, and inflammatory pathways. At the same time, scRNA-seq also detected some genes that had not been previously reported in cABMR. Taken together, these results suggest that changes in these DEGs may be involved in the pathogenesis of cABMR, and the significance and specificity of these markers need to be verified by further studies.

The extensive activation of T-, B-cells and neutrophils was observed in this scRNA-seq, which indicated that these biological processes may play an important role in cABMR. By analyzing the receptor-ligand interactions between different cell types in cABMR, we evaluated the intercellular signaling network. For example, chemokine response pathways, the CD94:NKG2A/C-HLA-E signaling axis, and the CD2-CD58 signaling axis were observed in T-cell subsets; all these signaling networks are involved in T cell activation. Interestingly, CD74-Copa and PTPRC-CD22 signaling axes were not only activated in B cells but also played an important role in signal transduction between T/B cells and neutrophils. CD74 plays an important role in regulating T- and B-cell development, dendritic cell (DC) movement, macrophage inflammation, and thymus selection. Furthermore, CD74 regulates the maturation of B cells through the NF-κB p65/RelA homodimer and its coactivator, TAFIII105 (46). Thus, CD74 may be involved in the occurrence of cABMR by regulating the maturation of B-cells through the CD74-COPA signaling axis.Furthermore, we reported for the first time that ANXA1-FPR1/2H, C5AR1-RPS19, and CCL5-CCR5 signal axes were involved in the occurrence of cABMR. CCR5 is expressed in a variety of cell types, including T cells, macrophages, dendritic cells, eosinophils, and microglia. It plays a fundamental role in the inflammatory response by directing cells to the site of inflammation. In cABMR, the CCL5/CCR5 axis may activate T-cell subsets in autocrine and paracrine ways and induce proinflammatory and rejection responses of T-cells. Thus, further confirmation of these interactions would be important for the development of novel therapeutic targets.

On the basis of single-cell sequencing, in order to further verify the discovery of single-cell sequencing, we collected additional samples from 9 control patients and 5 cABMR patients, and further verified abnormal gene expression in single-cell sequencing by western blot and qRT-PCR experiments. The results indicated that MT-ND6, CCL4L2, CXCR4, NFKBIZ, DUSP1 and ZFP36 were up-regulated in cABMR patients, and MAPK and NFκB signaling pathways were also confirmed to be activated. Thus, western blot and qRT-PCR experiments indicated that single-cell sequencing can be a reliable strategy for detailed understanding of peripheral blood lymphocyte differences in cABMR patients. This study also has some limitations. First of all, the sample size of this study is relatively small. Thus, in future studies, more clinical samples need to be enrolled for single-cell sequencing to reflect the severity and stage of the disease, and to limit the heterogeneity and individual differences of cABMR. Finally, our results are preliminary and require further systematic experimental validation and clinical patient evaluation based on the single-cell sequencing findings.

In conclusion, this study lays the foundation for a comprehensive multicellular description of the complex dynamic immune response of cABMR after renal transplantation. We also presented a cell-specific renal transcriptome and identified a series of novel genes involved in signaling pathways and potential ligand-receptor interactions in cABMR patients. The transcriptomic data, coupled with detailed scRNA-seq lineage information, can serve as a rich resource for a detailed understanding of peripheral lymphocytes in patients with cABMR and pave the way for the design of potential novel therapies.

## Data Availability Statement

The datasets presented in this study can be found in online repositories. The names of the repository/repositories and accession number(s) can be found below: https://www.ncbi.nlm.nih.gov/geo/query/acc.cgi?acc=GSE190329.

## Ethics Statement

Written informed consent was obtained from the individual(s) for the publication of any potentially identifiable images or data included in this article.

## Author Contributions

FK and SY contributed to the manuscript writing and data analysis. ZZ, XZ, WZ, ZL, and JL contributed to data collection and collation. YX and QY contributed to the design of the project and the revision of the manuscript. All authors contributed to the article and approved the submitted version.

## Funding

This study was supported by the National Natural Science Foundation of China, grant 81970548; Medical Science Advancement Program (Clinical Medicine) of Wuhan University, grant TFLC2018003 and Wuhan Science and Technology Project, grant 2019020701011485.

## Conflict of Interest

The authors declare that the research was conducted in the absence of any commercial or financial relationships that could be construed as a potential conflict of interest.

## Publisher’s Note

All claims expressed in this article are solely those of the authors and do not necessarily represent those of their affiliated organizations, or those of the publisher, the editors and the reviewers. Any product that may be evaluated in this article, or claim that may be made by its manufacturer, is not guaranteed or endorsed by the publisher.

## Supplementary Material

Supplementary Figure 1 The results of transplanted kidney biopsy in cABMR patients were based on Banff 2019 criteria: **(A)** Normal glomeruli and tubules; **(B)** Perirenal capillaritis of cABMR, lymphocytes infiltration (Black arrow); **(C)** glomerulitis of cABMR(Masson dyeing, black arrow); **(D)** Chronic glomerulonephrosis, basal membrane thickening, and bitrack changes(PASM dyeing, black arrow); **(E)** Multilayer basal membrane changes in renal peritubule capillaries(Transmission electron microscope, black arrow); **(F)** Perirenal capillaritis of cABMR, lymphocytes infiltration (Black arrow); **(G)** glomerulitis of cABMR(Masson dyeing, black arrow); **(H)** Chronic glomerulonephrosis, basal membrane thickening, and bitrack changes(PASM dyeing, black arrow).

Supplementary Figure 2 Quality control charts of 4 samples. **(A)** The distribution of samples in the control group was statistically plotted, and the distribution of various types of cells was shown in the form of violin distribution (From left to right: 1. Distribution proportion of mitochondrial gene content (percent. mito); 2. 2. Number of UMI in cells (nCount); 3. Number of genes in cells (nFeature)); **(B)** The distribution of samples in the cABMR group was statistically plotted, and the distribution of various types of cells was shown in the form of violin distribution(From left to right: 1. Distribution proportion of mitochondrial gene content (percent. mito); 2. 2. Number of UMI in cells (nCount); 3. Number of genes in cells (nFeature)).

Supplementary Figure 3 GO and KEGG enrichment analysis of T cell subsets. **(A)** GO enrichment analysis of down-regulated DEGs of CD8 effector T cells; **(B, C)** GO **(B)** and KEGG **(C)** enrichment analysis of down-regulated DEGs in CD8_MAI T cells; **(D, E)** GO(D) and KEGG **(E)** enrichment analysis of down-regulated DEGs in γδ T cells.

Supplementary Figure 4 GO and KEGG enrichment analysis of B cell subsets **(A, B)** GO(A) and KEGG(B) enrichment analysis of down-regulated DEGs in Naive B cells; **(C, D)** GO **(C)** and KEGG **(D)** enrichment analysis of down-regulated DEGs in Plasma cells.

Supplementary Table 1 Clinical and biochemical features of patients.

Supplementary Table 2 Cell types and corresponding marker genes.

Supplementary Table 3 List of Marker genes for T cells subtype subdivision.

Supplementary Table 4 DEGs in each T cell subsets from cABMR and control subjects shown in heatmap. Abbreviations were as follows: pct.1, the percentage of cells where the gene is detected in the first group (cABMR patients); pct.2, the percentage of cells where the gene is detected in the second group (control subject); avg logFC, log fold-change of the average expression between the two groups, positive values indicate that the gene is more highly expressed in the first group.

Supplementary Table 5 GO and KEGG enrichment in CD8 effector T cell subsets.

Supplementary Table 6 GO and KEGG enrichment in CD8_MAI T subsets.

Supplementary Table 7 GO and KEGG enrichment in γδ T cell subsets.

Supplementary Table 8 List of Marker genes for B cells subtype subdivision.

Supplementary Table 9 DEGs in each B cell subsets from cABMR and control subjects shown in heatmap.

Supplementary Table 10 GO and KEGG enrichment in Naive B cell subsets.

Supplementary Table 11 GO and KEGG enrichment in Plasma cell subsets.

Supplementary Table 12 ΔCt values of related genes in qRT-PCR experiment.
