# Supplementary figures and images for: Single-Cell Transcriptome Analysis of Chronic Antibody-Mediated Rejection After Renal Transplantation
Source: Front Immunol. 2022 Jan 17;12:767618. doi: 10.3389/fimmu.2021.767618 (PMC8801944; doi:10.3389/fimmu.2021.767618)

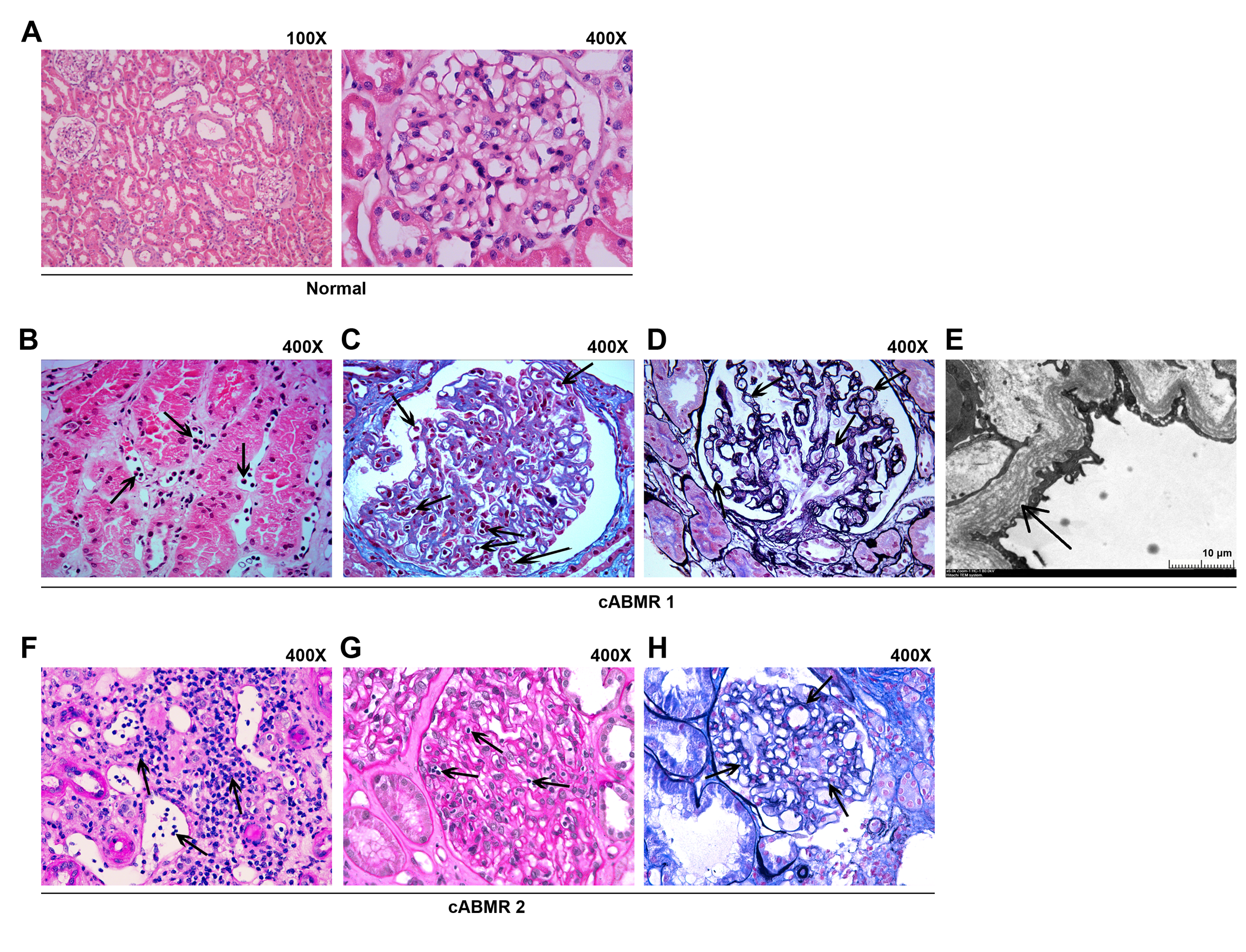

Supplement: Supplementary Figure 1 — The results of transplanted kidney biopsy in cABMR patients were based on Banff 2019 criteria: (A) Normal glomeruli and tubules; (B) Perirenal capillaritis of cABMR, lymphocytes infiltration (Black arrow); (C) glomerulitis of cABMR(Masson dyeing, black arrow); (D) Chronic glomerulonephrosis, basal membrane thickening, and bitrack changes(PASM dyeing, black arrow); (E) Multilayer basal membrane changes in renal peritubule capillaries(Transmission electron microscope, black arrow); (F) Perirenal capillaritis of cABMR, lymphocytes infiltration (Black arrow); (G) glomerulitis of cABMR(Masson dyeing, black arrow); (H) Chronic glomerulonephrosis, basal membrane thickening, and bitrack changes(PASM dyeing, black arrow). [file Image_1.tif]

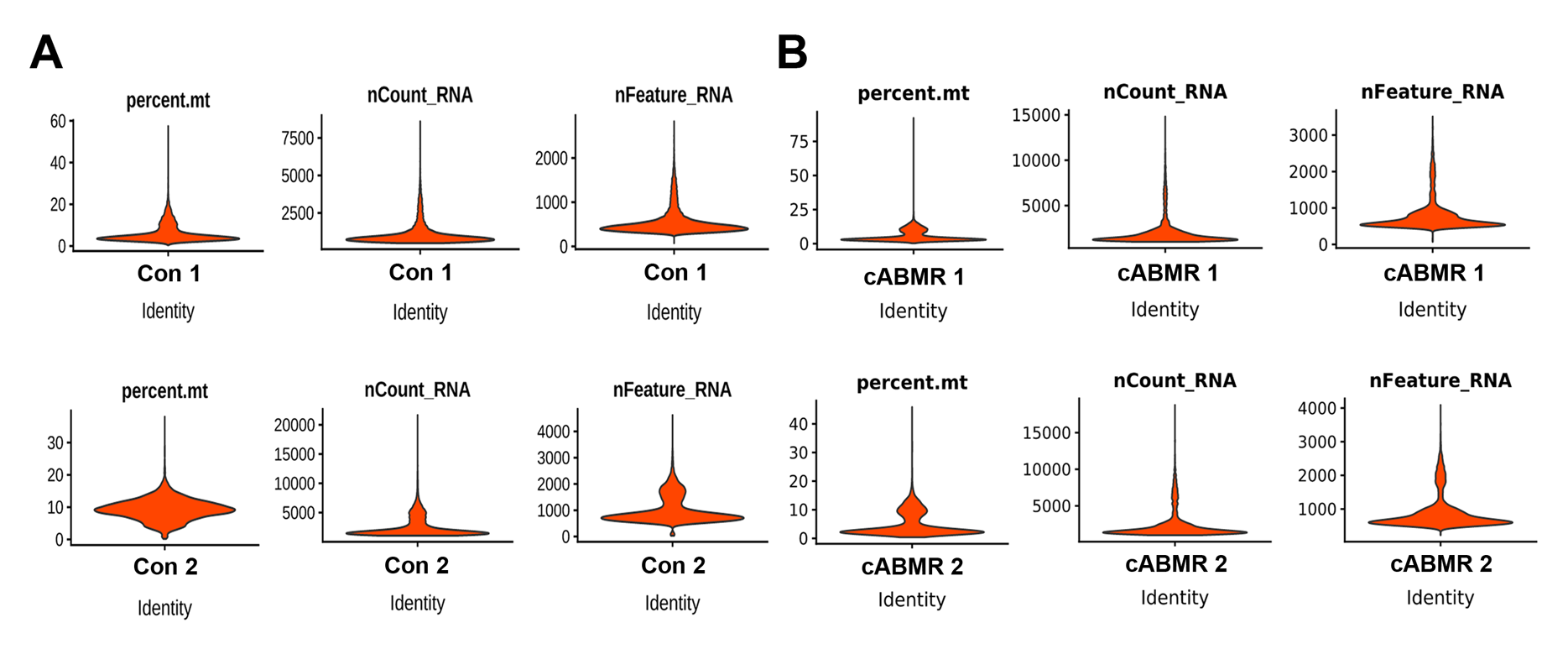

Supplement: Supplementary Figure 2 — Quality control charts of 4 samples. (A) The distribution of samples in the control group was statistically plotted, and the distribution of various types of cells was shown in the form of violin distribution (From left to right: 1. Distribution proportion of mitochondrial gene content (percent. mito); 2. 2. Number of UMI in cells (nCount); 3. Number of genes in cells (nFeature)); (B) The distribution of samples in the cABMR group was statistically plotted, and the distribution of various types of cells was shown in the form of violin distribution(From left to right: 1. Distribution proportion of mitochondrial gene content (percent. mito); 2. 2. Number of UMI in cells (nCount); 3. Number of genes in cells (nFeature)). [file Image_2.tif]

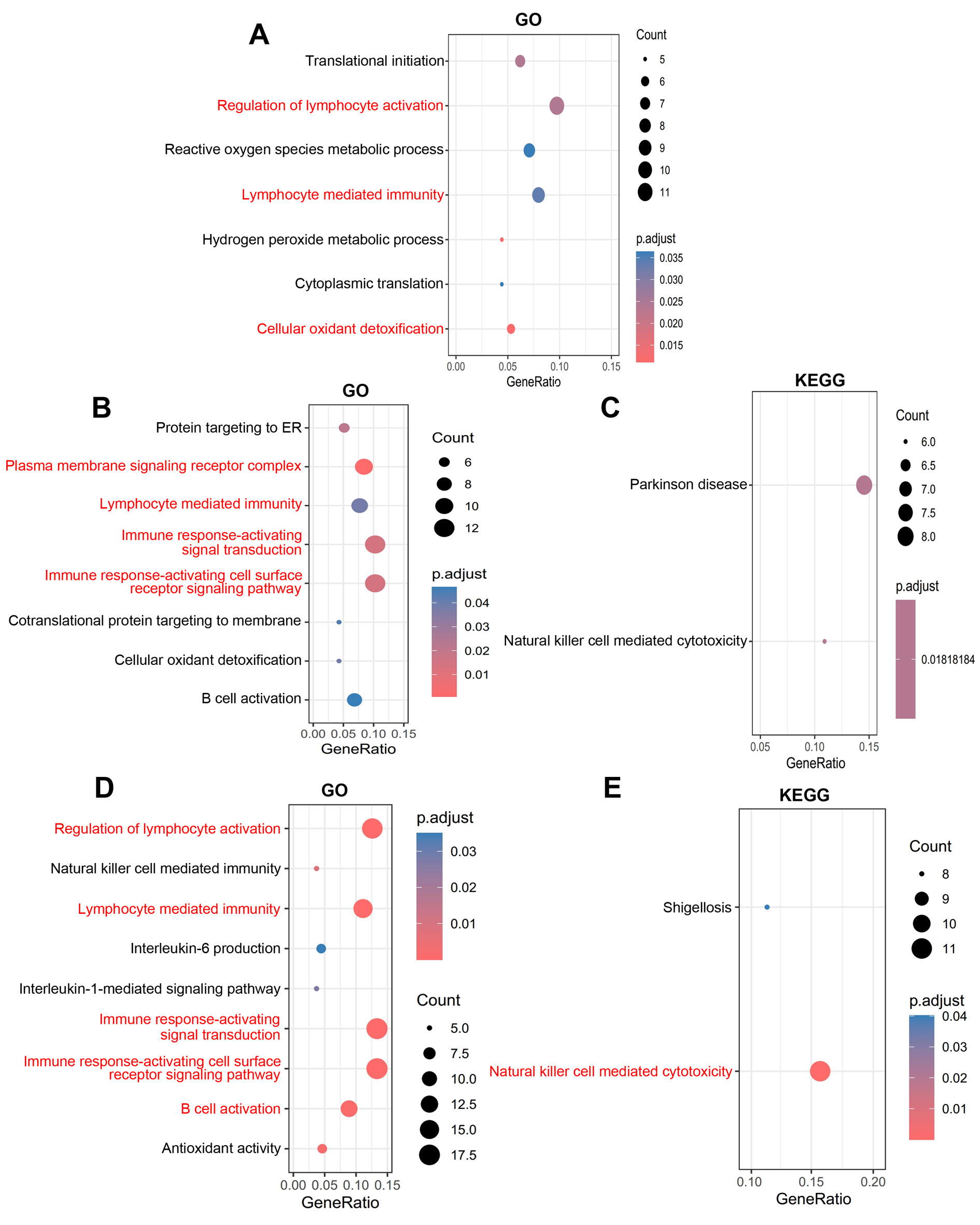

Supplement: Supplementary Figure 3 — GO and KEGG enrichment analysis of T cell subsets. (A) GO enrichment analysis of down-regulated DEGs of CD8 effector T cells; (B, C) GO (B) and KEGG (C) enrichment analysis of down-regulated DEGs in CD8_MAI T cells; (D, E) GO(D) and KEGG (E) enrichment analysis of down-regulated DEGs in γδ T cells. [file Image_3.tif]

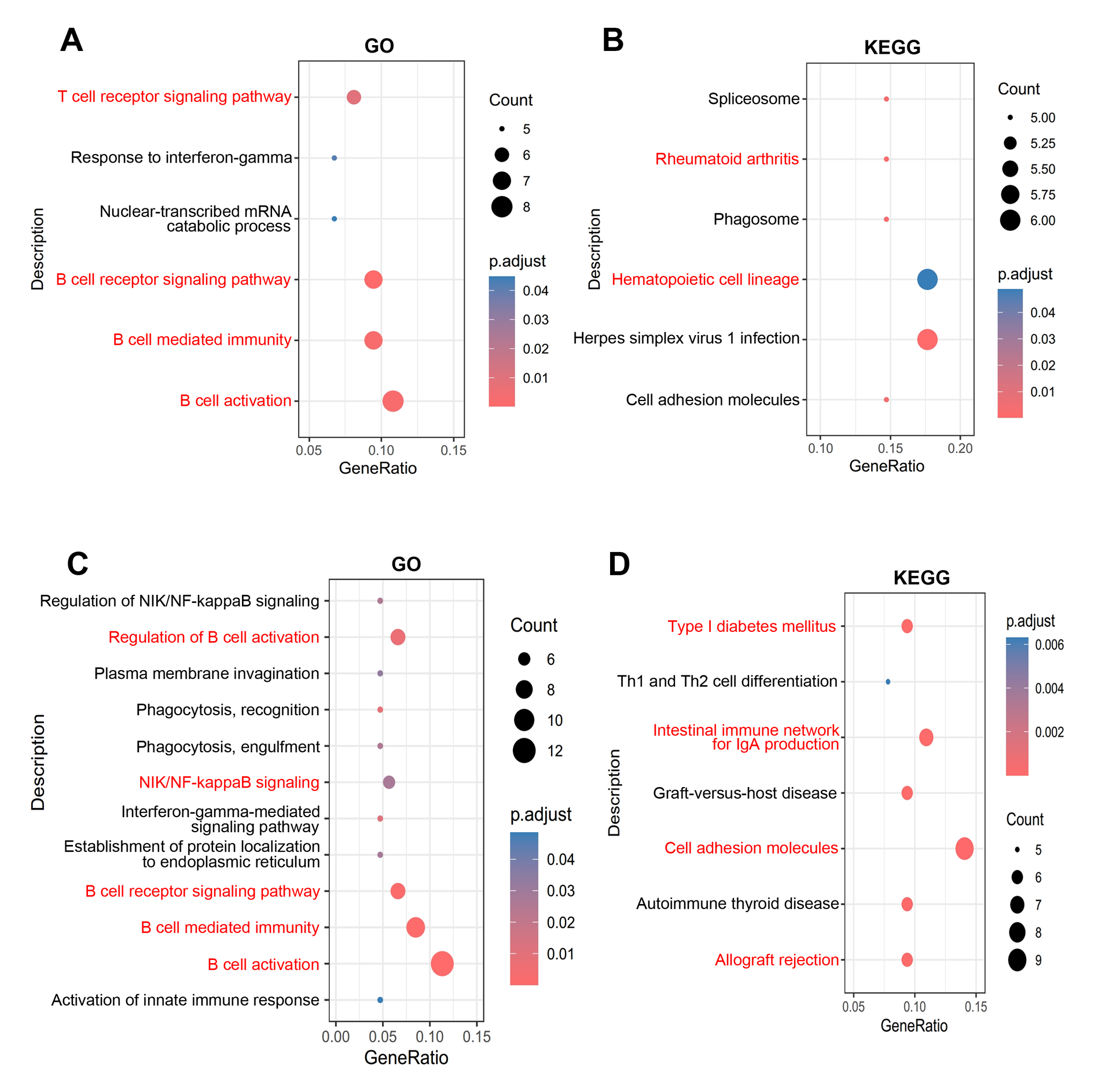

Supplement: Supplementary Figure 4 — GO and KEGG enrichment analysis of B cell subsets (A, B) GO(A) and KEGG(B) enrichment analysis of down-regulated DEGs in Naive B cells; (C, D) GO (C) and KEGG (D) enrichment analysis of down-regulated DEGs in Plasma cells. [file Image_4.tif]
