# Supplementary material for: Single-Cell Transcriptome Analysis of Chronic Antibody-Mediated Rejection After Renal Transplantation
Source: Front Immunol. 2022 Jan 17;12:767618. doi: 10.3389/fimmu.2021.767618 (PMC8801944; doi:10.3389/fimmu.2021.767618)
Supplement: Supplementary Table 1 — Clinical and biochemical features of patients. [file Table_1.docx]

Supplemental table 1: Clinical and biochemical features of patients

| Patients | Age(years) | Gender | Routine Urine | | | Renal Function | | | | | | | DSA | | |
| --- | --- | --- | --- | --- | --- | --- | --- | --- | --- | --- | --- | --- | --- | --- | --- |
|  |  |  | **Urine Protein** | **Leukocyte** | | **BUN**  **(mmol/L)** | **CREA**  **(μmol/L)** | | | | | **UA**  **(μmol/L)** | **PRA-Ⅰ** | **PRA-Ⅱ** | **PRA-total** |
| Control 1 | 37 | M | (-) | | (-) | 7.20 | | 159.3 | | | 348.3 | | (-) | (-) | (-) |
| Control 2 | 32 | M | (-) | | (-) | 7.9 | | 143.8 | | | 308.1 | | (-) | (-) | (-) |
| cABMR 1 | 30 | M | (+++) | | (-) | 18.31 | | 356.8 | | | 413.6 | | (-) | (-) | (-) |
| cABMR 2 | 35 | M | (+) | | (-) | 8.00 | | | 225.3 | 506.2 | | | (-) | (-) | (-) |
| P1 | 66 | M | (-) | | (-) | 12.35 | | | 120.43 | 301.21 | | | (+) | (-) | (+) |
| P2 | 53 | F | (-) | | (-) | 9.38 | | | 122.48 | 257.30 | | | (+) | (-) | (+) |
| P3 | 51 | M | (-) | | (-) | 10.71 | | | 117.38 | 311.25 | | | (-) | (-) | (-) |
| P4 | 53 | M | (-) | | (-) | 6.22 | | | 160.47 | 376.39 | | | (-) | (+++) | (++) |
| P5 | 35 | M | (+) | | (-) | 8.94 | | | 133.59 | 348.17 | | | (-) | (-) | (-) |
| P6 | 38 | M | (-) | | (-) | 11.29 | | | 127.45 | 270.41 | | | (-) | (-) | (-) |
| P7 | 43 | M | (-) | | (-) | 7.09 | | | 153.86 | 398.26 | | | (-) | (-) | (-) |
| P8 | 39 | F | (-) | | (-) | 7.82 | | | 149.58 | 344.72 | | | (+) | (-) | (+) |
| P9 | 30 | M | (-) | | (-) | 9.65 | | | 170.21 | 275.93 | | | (-) | (-) | (-) |
| P10 | 34 | M | (+) | | (+) | 20.74 | | | 238.40 | 603.44 | | | (-) | (-) | (-) |
| P11 | 42 | M | (++) | | (+) | 17.40 | | | 401.24 | 539.71 | | | (+) | (-) | (+) |
| P12 | 28 | M | (+) | | (++) | 15.66 | | | 387.92 | 602.83 | | | (-) | (-) | (-) |
| P13 | 45 | M | (+++) | | (+) | 18.83 | | | 324.77 | 547.06 | | | (-) | (-) | (+++) |
| P14 | 45 | F | (++) | | (-) | 16.39 | | | 355.09 | 631.84 | | | (+) | (+++) | (+++) |

(BUN: blood urea nitrogen; CREA: creatinine; UA: Uric Acid; PRA: panel reactive antibodies; DSA: donor specific antibody. (-): negative; (+): weakly positive; (++): positive; (+++): strongly positive)
