# Supplementary material for: Single-Cell Transcriptome Analysis of Chronic Antibody-Mediated Rejection After Renal Transplantation
Source: Front Immunol. 2022 Jan 17;12:767618. doi: 10.3389/fimmu.2021.767618 (PMC8801944; doi:10.3389/fimmu.2021.767618)
Supplement: Supplementary Table 2 — Cell types and corresponding marker genes. [file Table_2.docx]

| cell type | abbreviation | marker genes |
| --- | --- | --- |
| T cells | T cells | CD3D, CD3E, TRBC1 |
| NK cells | NK cells | CD3D-, KLRD1, NKG7, KLRC1, FCGR3A |
| B cells | B cells | MS4A1, CD79A, CD79B |
| Neutrophils | Neutrophils | LYZ, CSF3R, CXCR2, FCGR3B |
| Neutrophil progenitor cells | Pro-Neutrophil | CAMP, LTF, LCN2, MPO, AZU1 |
| Classical monocytes | Classical_mono | LYZ, CD14, FCN1, VCAN, FCGR3A |
| Non-Classical monocytes | Non-Classical mono | LYZ, FCN1, FCGR3A, CSF1R, CDKN1C |
| Dendritic cells | DCs | CD1C, CD1E, FCER1A, IL3RA, CLEC4C, LILRB4 |
| Basophils | Basophils | CLC, GATA2, CPA3, MS4A2 |
| Patelets | Patelets | PPBP, PF4, TUBB1 |

Supplemental table 2: Cell types and corresponding marker genes
