# Supplementary material for: Single-Cell Transcriptome Analysis of Chronic Antibody-Mediated Rejection After Renal Transplantation
Source: Front Immunol. 2022 Jan 17;12:767618. doi: 10.3389/fimmu.2021.767618 (PMC8801944; doi:10.3389/fimmu.2021.767618)
Supplement: Supplementary Table 3 — List of Marker genes for T cells subtype subdivision. [file Table_3.docx]

| Cell Type | Abbreviation | Marker Genes |
| --- | --- | --- |
| Naïve T cells/central memory T cells | Naïve T cells or Tcm | CD3D, CCR7, LEF1, SELL, TCF7, IL7R |
| CD8+  effector T cells | - | CD3D, CD8A/B, NKG7, GZMA, GNLY |
| γδ T cells+CD8+ mucosal-associated invariant T cells | γδ T | CD3D, TRDC, TRGC1, TRGC2 |
| CD8+ mucosal-associated invariant T cells | CD8+ MAIT | CD3D, CD8A, SLC4A10, KLRB1 |

Supplemental table 3: List of Marker genes for T cells subtype subdivision
