# Supplementary material for: Single-Cell Transcriptome Analysis of Chronic Antibody-Mediated Rejection After Renal Transplantation
Source: Front Immunol. 2022 Jan 17;12:767618. doi: 10.3389/fimmu.2021.767618 (PMC8801944; doi:10.3389/fimmu.2021.767618)
Supplement: Supplementary Table 8 — List of Marker genes for B cells subtype subdivision. [file Table_8.docx]

Supplemental table 8: List of Marker genes for B cells subtype subdivision

| Cell Type | Marker Genes |
| --- | --- |
| Naïve B cells | MS4A1, TCL1A, IGHD, IL4R, IGHM |
| Plasma cells | JCHAIN, IGHA1, IGHG1 |
