# Supplementary material for: Single-Cell Transcriptome Analysis of Chronic Antibody-Mediated Rejection After Renal Transplantation
Source: Front Immunol. 2022 Jan 17;12:767618. doi: 10.3389/fimmu.2021.767618 (PMC8801944; doi:10.3389/fimmu.2021.767618)
Supplement: Supplementary Table 12 — ΔCt values of related genes in qRT-PCR experiment. [file Table_12.docx]

| Genes | Control_(P1-P9)_ | | | | | | | | | | cABMR_(P10-P14)_ | | | | | |
| --- | --- | --- | --- | --- | --- | --- | --- | --- | --- | --- | --- | --- | --- | --- | --- | --- |
|  | P1 | P2 | P3 | P4 | P5 | P6 | P7 | P8 | P9 | P10 | | P11 | P12 | P13 | P14 |  |
| CCL4L2 | 9.56 | 12.47 | 10.98 | 11.23 | 15.62 | 10.59 | 14.33 | 15.24 | 14.32 | 8.11 | | 7.15 | 10.87 | 11.05 | 7.52 |  |
| CXCL8 | 10.03 | 8.14 | 15.98 | 12.47 | 13.66 | 9.23 | 11.27 | 9.93 | 11.34 | 6.19 | | 9.64 | 10.35 | 8.64 | 7.88 |  |
| CXCR4 | 10.29 | 9.35 | 13.44 | 8.37 | 11.18 | 13.67 | 15.26 | 8.62 | 14.13 | 8.18 | | 9.72 | 7.06 | 10.46 | 7.5 |  |
| DUSP1 | 9.87 | 13.58 | 14.06 | 11.32 | 10.69 | 14.57 | 17.69 | 9.26 | 10.49 | 7.28 | | 10.09 | 6.17 | 9.33 | 8.1 |  |
| FOS | 12.58 | 13.64 | 10.29 | 11.69 | 7.14 | 8.37 | 15.52 | 13.06 | 9.81 | 5.35 | | 10.24 | 9.63 | 7.08 | 8.35 |  |
| JUN | 17.69 | 15.24 | 13.11 | 16.64 | 13.29 | 14.08 | 15.33 | 16.35 | 13.2 | 12.34 | | 16.08 | 9.62 | 11.47 | 12.35 |  |
| MT-ND6 | 16.22 | 14.18 | 13.2 | 17.63 | 15.69 | 12.34 | 15.98 | 14.82 | 16.38 | 12.85 | | 15.37 | 10.33 | 9.68 | 10.23 |  |
| NFKBIA | 19.32 | 17.66 | 15.43 | 16.87 | 14.05 | 16.18 | 13.64 | 18.99 | 17.13 | 15.38 | | 16.06 | 13.22 | 10.08 | 12.82 |  |
| NFKBIZ | 15.07 | 11.98 | 16.44 | 19.02 | 15.29 | 16.55 | 17.48 | 16.3 | 18.43 | 13.39 | | 10.02 | 14.88 | 15.74 | 11.62 |  |
| S100A9 | 10.25 | 19.32 | 16.24 | 17.59 | 18.33 | 16.53 | 14.2 | 16.05 | 17.34 | 15.29 | | 11.44 | 11.97 | 8.07 | 9.21 |  |
| TNFAIP3 | 19.35 | 9.84 | 15.63 | 15.35 | 14.92 | 10.38 | 18.64 | 17.99 | 16.59 | 6.38 | | 9.64 | 16.21 | 12.05 | 8.11 |  |
| ZFP36 | 14.08 | 10.22 | 10.94 | 15.39 | 10.28 | 11.96 | 15.37 | 15.93 | 18.08 | 6.74 | | 10.28 | 9.07 | 8.91 | 13.82 |  |

Supplemental table 12: △Ct values of related genes in qRT-PCR experiment
